# Associations of parental and perinatal factors with subsequent risk of stress-related disorders: a nationwide cohort study with sibling comparison

**DOI:** 10.1038/s41380-021-01406-5

**Published:** 2022-01-01

**Authors:** Yuchen Li, Arvid Sjölander, Huan Song, Sven Cnattingius, Fang Fang, Qian Yang, Lorena Fernández de la Cruz, David Mataix-Cols, Gustaf Brander, Jiong Li, Wei Zhang, Katja Fall, Brian M. D’Onofrio, Catarina Almqvist, Paul Lichtenstein, Unnur A. Valdimarsdóttir, Donghao Lu

**Affiliations:** 1 Mental Health Center, West China Hospital of Sichuan University, Chengdu, China; 2 West China Biomedical Big Data Center, West China Hospital, Sichuan University, Chengdu, China; 3 Department of Medical Epidemiology and Biostatistics, Karolinska Institutet, Stockholm, Sweden; 4 Center of Public Health Sciences, Faculty of Medicine, University of Iceland, Reykjavík, Iceland; 5 Division of Clinical Epidemiology, Department of Medicine Solna, Karolinska University Hospital, Karolinska Institutet, Stockholm, Sweden; 6 Institute of Environmental Medicine, Karolinska Institutet, Stockholm, Sweden; 7 Department of Clinical Neuroscience, Centre for Psychiatry Research, Karolinska Institutet, Stockholm, Sweden; 8 Stockholm Health Care Services, Region Stockholm, Stockholm, Sweden; 9 Department of Clinical Medicine-Department of Clinical Epidemiology, Aarhus University, Aarhus, Denmark; 10 Clinical Epidemiology and Biostatistics, School of Medical Sciences, Örebro University, Örebro, Sweden; 11 Department of Psychological and Brain Sciences, Indiana University, Bloomington, USA; 12 Astrid Lindgren Children’s Hospital, Karolinska University Hospital, Stockholm, Sweden; 13 Department of Epidemiology, Harvard T.H. Chan School of Public Health, Boston, MA USA

**Keywords:** Diseases, Psychiatric disorders

## Abstract

Little is known about the contribution of pregnancy-related parental and perinatal factors to the development of stress-related disorders. We aimed to investigate whether parental/perinatal adversities entail higher risks of stress-related disorders in the offspring, later in life, by accounting for genetic and early environmental factors. Based on the nationwide Swedish registers, we conducted a population-based cohort study of 3,435,747 singleton births (of which 2,554,235 were full siblings), born 1973–2008 and survived through the age of 5 years. Using both population- and sibling designs, we employed Cox regression to assess the association between parental and perinatal factors with subsequent risk of stress-related disorders. We identified 55,511 individuals diagnosed with stress-related disorders in the population analysis and 37,433 in the sibling analysis. In the population-based analysis we observed increased risks of stress-related disorders among offspring of maternal/paternal age <25, single mothers, parity ≥4, mothers with BMI ≥ 25 or maternal smoking in early pregnancy, gestational diabetes, and offspring born moderately preterm (GA 32–36 weeks), or small-for-gestational-age. These associations were significantly attenuated toward null in the sibling analysis. Cesarean-section was weakly associated with offspring stress-related disorders in population [hazard ratio (HR) 1.09, 95% confidence interval (CI) 1.06–1.12] and sibling analyses (HR 1.10, 95% CI 1.02–1.20). Our findings suggest that most of the observed associations between parental and perinatal factors and risk of stress-related disorders in the population analysis are driven by shared familial environment or genetics, and underscore the importance of family designs in epidemiological studies on the etiology of psychiatric disorders.

## Introduction

Most people are exposed to some stressful life events or trauma throughout the life span [1]. Such events raise the risk of severe psychiatric reactions, including posttraumatic stress disorder (PTSD) and acute stress reaction (ASR), adjustment disorder, and other stress reactions [1, 2]. These stress-related disorders are associated with a number of adverse health consequences, including cardiovascular [3] and metabolic diseases [4], autoimmune diseases [5], dementia [6], suicide [7], and premature mortality [8]. Some risk factors have been identified, including female sex, socioeconomic disadvantage, history of chronic or major somatic illness, individual and family history of psychiatric disorders, and trauma severity, which have all been associated with stress-related disorders [9–11]. The prevalence of PTSD is relatively stable from adolescence through midlife [12]. However, except for childhood traumatic experiences [13], little is known about potential risk factors occurring before or shortly after birth [14].

Animal models have shown that parental factors during pregnancy and perinatal stressors may influence the development of early psychopathology [15]. Emerging evidence also supports that adverse parental and perinatal factors may be associated with a range of psychiatric disorders in humans [16–18]. For instance, individuals with low birth weight have been reported to be at higher risks for depression [16] and obsessive-compulsive disorder [17], whereas preterm birth is associated with increased risks of obsessive-compulsive disorder [17], tic disorders [18] and suicide attempt [19]. To date, it is unknown whether adverse parental or perinatal factors are associated with subsequent risk of stress-related disorders. Familial factors, e.g., genetics and socioeconomic status, may account for the association between parental and/or perinatal exposures and offspring’s outcomes [20, 21], highlighting the need of family-based designs, e.g., using sibling comparison, in this field.

Leveraging a nationwide population birth cohort in Sweden, we aimed to identify parental and perinatal factors associated with risk of stress-related disorders later in life. To disentangle the familial influence on the studied associations, we also performed a sibling analysis [20].

## Materials and methods

### Study population

Every Swedish resident is assigned a unique personal identity number (PIN) at birth or at immigration, which is used for data linkage across registers. Since 1973, the Swedish Medical Birth Register (MBR) has collected information on >98% of deliveries in Sweden, starting from women’s first visit to prenatal care, through delivery and birth care [22]. Information includes mothers’ and infants’ PINs, maternal characteristics, mode of delivery, pregnancy complications, and birth outcomes. Based on this register, we identified 3,546,149 singleton live births from January 1st, 1973 to December 31st, 2008. We excluded stillbirths (*n* = 13,365) and individuals without information on PIN (*n* = 5041), gestational age (*n* = 8323) or sex (*n* = 1). Because stress-related disorders are usually not diagnosed in individuals below 5 years of age [23], we followed all individuals from their 5th birthday. Therefore, we also excluded 83,672 individuals who either died or emigrated before age 5, leaving 3,435,747 individuals for the population analysis. By linking to the Migration [24] and Cause of Death Registers [25], the follow-up was censored at first emigration, death or December 31st, 2013, whichever came first.

To identify full siblings, we obtained information on biological fathers through linkage to the Multi-Generation Register (MGR), while maternal PINs are already documented in the MBR. The MGR documents parental information on Swedish residents that were born from 1932 onward and were alive since 1961 [26]. In total, we identified 2,554,235 (74.3%) individuals having at least one full sibling (i.e., sharing the same biological mother and father) for the sibling analyses.

### Ascertainment of parental and perinatal factors

We defined factors originating from early pregnancy which are related to the parents as parental factors and factors pertaining to the time around birth as perinatal factors. We obtained information on parental and perinatal factors from the MBR, while the National Patient Register was used to complement diagnostic information on maternal hypertensive, diabetic, and psychiatric disorders (see Supplementary Table 1 for diagnostic codes).

#### Parental factors

Information on maternal age at childbirth and maternal relationship status (i.e., living together or not) with the offspring’s father was extracted from the MBR, while paternal age at childbirth was calculated from the MGR. Information on parity (including the present birth) was extracted from the MBR and categorized as 1,2,3, ⩾4 (the birth of fourth child or thereafter). Maternal weight and height at early pregnancy were recorded at the first parental visit (usually at 12 gestational weeks) [27] from 1992 onward. Maternal body mass index (BMI) was calculated and classified as <18.5, 18.5 to <25 (reference), 25 to <30, and ⩾30 kg/m^2^. Maternal smoking was documented at the first parental visit from 1982 onward, and grouped as no smoking, 1–9, and ≥10 cigarettes per day [28]. We also studied common pregnancy complications, such as maternal hypertensive (i.e., preeclampsia and essential hypertension) and diabetic diseases (i.e., gestational diabetes and pregestational diabetes; information is available from 1987 onward). Analyses of maternal BMI, smoking, and diabetes were restricted to individuals born after 1992, 1982, and 1987, respectively.

#### Perinatal factors

Mode of delivery was categorized as cesarean section, instrumental vaginal delivery (forceps or vacuum extraction), and non-instrumental vaginal delivery. Gestational age was primarily estimated from the results of ultrasonography performed early in the second trimester, which was introduced in Sweden in the late 1970s and was offered to all pregnant women since 1990 [29]. Gestational age was categorized as very preterm (gestational age <32 completed weeks), moderately preterm (32–36 weeks), term (37–41 weeks), and post-term birth (≥42 weeks). We used the ultrasound-based, sex-specific Swedish reference curve for fetal growth [30] to derive birth weight for gestational age percentiles and categorized it as <3rd, 3rd to <10th, 10th to 90th, >90th to 97th, and >97th percentile. The Apgar score is a quick assessment for the newborn’s vital status, by evaluating heart rate, respiratory effort, reflex irritability, muscle tone, and color after delivery [31]. Low 5 min Apgar score was defined as <7 at 5 min.

### Ascertainment of stress-related disorders

We defined stress-related disorders as PTSD, ASR, adjustment disorder, and other stress reactions. Through linkage to the National Patient Register, we identified any first inpatient hospital or outpatient specialist visit with a stress-related disorder as the main diagnosis, using the codes of the 8–10th Swedish versions of International Classification of Disease (ICD-8: 307, 308.4; ICD-9: 308, 309; and ICD-10: F43; listed in Supplementary Table 1). The National Patient Register covers 86% psychiatric diagnoses of the entire country from 1973, and became nationwide from 1987 [32]. From 2001, the Register also collected the hospital-based outpatient visits (initial coverage >80%) [32].

### Covariates

Information on covariates was primarily extracted from the MBR and the MGR, including maternal country of birth (Nordic or non-Nordic countries; the former includes Denmark, Finland, Iceland, Norway, and Sweden), offspring year of birth, and sex. We assessed history of parental (both maternal and paternal) psychiatric disorders by using any diagnosis of a psychiatric disorder, identified from the National Patient Register, before the pregnancy. As a proxy for socioeconomic status, we obtained the highest educational level of the mother from the Education Register.

### Statistical analysis

First, we estimated the crude incidence rates (IRs) of stress-related disorders by baseline characteristics. In the primary analysis, we calculated crude IRs and hazard ratios (HRs) with 95% confidence intervals (CIs) of stress-related disorders for each parental and perinatal factor in the population analysis. We used Cox regression with attained age as the underlying timescale, stratified by year of birth in groups and adjusted for offspring sex, maternal relationship status, maternal country of birth, maternal highest educational level, and history of parental psychiatric disorders. To address confounding by familial factors, we employed Cox regression stratified by sibling sets and adjusted for offspring sex, year of birth (in groups), and maternal relationship status. We described numbers of families and siblings discordant on studied parental/perinatal factors and stress-related disorders in Supplementary Table 2. We did not perform sibling analyses for maternal chronic diseases (i.e., pregestational diabetes and essential hypertension), because all siblings (i.e., no exposure variation between these siblings). We tested the proportional hazard assumption using the Schoenfeld residual test and found no violation for any of the covariates [33].

To better control for the maternal background factors in the population analysis, we applied a finer adjustment for maternal country of birth (Sweden, other Nordic, European, African, Asian, or other countries) in an additional analysis. To increase the comparability between results from the population and sibling cohorts, we applied the population analysis by restricting to individuals with at least one sibling. To shed light on independent effects, we also performed an analysis by mutually adjusting for parental or perinatal factors, respectively.

Individuals with stress-related disorders often have other comorbid psychiatric disorders (e.g., depression, anxiety, substance abuse) [34], which may drive the observed associations. We therefore identified psychiatric comorbidities for all individuals as any inpatient or outpatient visit with a main diagnosis of a psychiatric disorder other than stress-related disorders during the follow-up from the National Patient Register (see diagnostic codes in Supplementary Table 1). To test whether the increased risk of stress-related disorders was driven by psychiatric comorbidities, we conducted a sensitivity analysis by restricting the cohort to individuals with stress-related disorders without psychiatric comorbidities, either before or after the diagnosis of stress-related disorder. To shed light on the subtype of more severe stress-related disorders, we performed separate analyses for PTSD and ASR.

The data were prepared in SAS statistical software, version 9.4 (SAS Institute, Cary, NC) and analyzed in Stata 15.1 (STATA, College Station, TX). The study was approved by the Regional Ethics Review Board in Stockholm, Sweden (reference number 2013/862–31/5). All data were pseudonymized prior to analyses. Written consent was not required given the nature of population registry resources.

## Results

### Baseline characteristics

During the long-term follow-up up to 35 years (median 17.4 years), we identified 55,511 individuals diagnosed with stress-related disorders for the population analysis and 37,433 for the sibling analysis. The mean age at diagnosis was 24.2 years (standard deviation, 6.9 years). The crude IR of stress-related disorders was higher among individuals born before 1997, in females, in offspring of mothers with lower educational attainment, not living with a partner, and when parents had a history of psychiatric disorders before pregnancy (Table 1).

**Table 1:** Baseline characteristics of individuals included in the population and sibling analyses.

	Population analysis			Sibling analysis		
	Individuals, *N*	Cases, *N*	IR	Individuals, *N*	Cases, *N*	IR
Total number	3,435,747	55,511	0.9	2,554,235	37,433	0.8
Year of birth						
1973–1977	488,419	13,753	0.9	267,878	6965	0.8
1978–1981	358,665	9998	1.0	274,943	6924	0.9
1982–1986	449,711	12,127	1.1	369,997	9074	1.0
1987–1991	547,090	11,442	1.1	455,073	8647	1.0
1992–1996	512,842	6244	0.8	418,717	4532	0.7
1997–2001	412,063	1603	0.4	331,068	1086	0.3
2002–2008	666,957	344	0.2	436,559	205	0.1
Offspring sex						
Female	1,669,979	35,506	1.2	1,240,080	24,076	1.1
Male	1,765,768	20,005	0.6	1,314,155	13,357	0.6
Maternal country of birth						
Nordic	3,091,355	51,852	0.9	2,319,684	34,978	0.8
Non-Nordic	343,328	3654	0.9	234,488	2455	0.8
Unknown	1064	5	0.8	63	0	0
Maternal education, years						
<10	432,679	11,262	1.2	280,929	6762	1.2
10–12	1,632,372	28,769	1.0	1,209,511	19,387	0.9
⩾13	1,347,481	15,019	0.7	1,054,163	11,077	0.7
Unknown	23,215	461	1.4	9632	207	1.5

*N* number, *IR* incidence rate (per 1000 person-years in crude).

### Parental factors

In the population analysis, we observed increased risks of stress-related disorders among offspring of younger mothers and fathers (≤24 years), older mothers (⩾35 years), not cohabiting mothers, parity ⩾ 4, history of parental psychiatric disorders before pregnancy, mothers with BMI ⩾ 25 kg/m^2^ in early pregnancy, smoking in early pregnancy, and mothers with essential hypertension or gestational diabetes. However, all these associations were attenuated toward null in the sibling analyses (Table 2). For instance, maternal smoking associated HRs (95% CIs) were in population analyses 1.45 (1.41–1.50) for 1–9 cigarettes per day and 1.66 (1.61–1.72) for ⩾10 cigarettes per day, while corresponding HRs (95% CIs) in sibling analyses attenuated to 1.06 (0.97–1.16) and 1.04 (0.93–1.16), respectively.

**Table 2:** Associations of parental factors with subsequent risk of stress-related disorders.

	Population analysis^a^			Sibling analysis^b^		
	Individuals, *N*	Cases, *N* (IR)	HR (95% CI)	Individuals, *N*	Cases, *N* (IR)	HR (95% CI)
Maternal age at childbirth, years						
<20	113,570	4153 (1.7)	1.80 (1.74–1.86)	65,365	2234 (1.5)	1.04 (0.93–1.17)
20–24	730,053	16,301 (1.1)	1.24 (1.21–1.27)	550,006	11,439 (1.0)	0.99 (0.93–1.05)
25–29	1,223,891	18,508 (0.8)	1.00	955,914	13,415 (0.8)	1.00
30–34	933,024	11,216 (0.8)	0.97 (0.95–0.99)	700,410	7472 (0.7)	1.02 (0.95–1.09)
⩾35	435,209	5333 (0.9)	1.07 (1.04–1.10)	282,540	2873 (0.8)	1.02 (0.91–1.16)
Paternal age at childbirth, years						
<20	25,560	982 (1.9)	1.90 (1.78–2.03)	12,051	452 (1.7)	1.18 (0.98–1.41)
20–24	379,304	9881 (1.2)	1.31 (1.28–1.35)	268,507	6418 (1.1)	1.05 (0.98–1.11)
25–29	1,044,087	18,022 (0.9)	1.00	813,199	13,024 (0.8)	1.00
30–34	1,081,295	14,459 (0.8)	0.92 (0.90–0.95)	835,673	10,270 (0.7)	0.99 (0.94–1.05)
⩾35	881,781	11,572 (0.9)	1.00 (0.98–1.03)	623,656	7242 (0.8)	0.98 (0.88–1.09)
Maternal cohabitation status						
Non-cohabitation	404,794	11,948 (1.2)	1.36 (1.33–1.39)	226,851	6079 (1.0)	0.99 (0.93–1.05)
Cohabitation	2,769,214	38,388 (0.9)	1.00	2,133,886	27,883 (0.8)	1.00
Parity						
1	1,459,407	23,310 (0.9)	1.00	958,719	14,326 (0.8)	1.00
2	1,264,262	18,992 (0.9)	0.91 (0.90–0.93)	1,042,972	13,961 (0.8)	0.93 (0.89–0.97)
3	508,833	8763 (1.0)	0.99 (0.97–1.01)	394,508	6023 (0.9)	0.88 (0.81–0.95)
⩾4	203,245	4446 (1.3)	1.21 (1.18–1.26)	158,036	3123 (1.2)	0.80 (0.71–0.91)
Maternal BMI at early pregnancy, kg/m^2c^						
<18.5	32,223	173 (0.6)	1.04 (0.91–1.20)	22,588	132 (0.6)	0.74 (0.43–1.28)
18.5 to <25	804,551	3180 (0.5)	1.00	596,592	2228 (0.4)	1.00
25 to <30	307,358	1296 (0.6)	1.14 (1.07–1.20)	227,532	923 (0.5)	1.01 (0.80–1.27)
⩾30	124,430	570 (0.7)	1.35 (1.24–1.46)	90,496	407 (0.6)	0.94 (0.66–1.35)
Maternal smoking during early pregnancy^d^						
No smoking	1,953,142	17936 (0.7)	1.00	1,556,424	14,282 (0.7)	1.00
1–9 cigarettes per day	284,981	6071 (1.3)	1.45 (1.41–1.50)	201,086	4091 (1.2)	1.06 (0.97–1.16)
⩾10 cigarettes per day	159,654	4588 (1.7)	1.66 (1.61–1.72)	107,883	2907 (1.5)	1.04 (0.93–1.16)
Maternal hypertensive diseases						
No	3,322,341	53,887 (0.9)	1.00	2,476,248	36,412 (0.8)	1.00
Preeclampsia	92,693	1377 (0.9)	1.02 (0.96–1.07)	63,036	848 (0.8)	1.06 (0.94–1.19)
Essential hypertension	20,713	247 (1.0)	1.15 (1.02–1.31)	–	–	–
Maternal diabetes^e^						
No	2,086,953	19,299 (0.8)	1.00	1,603,650	14,241 (0.7)	1.00
Gestational diabetes	18,853	184 (0.9)	1.15 (0.99–1.32)	13,615	127 (0.9)	0.66 (0.46–0.96)
Pregestational diabetes	13,513	150 (1.0)	1.18 (1.01–1.39)	–	–	–

Note: Individuals with missing information on paternal age at childbirth (*N* = 23,720, 0.69%), maternal cohabitation status (*N* = 261,739, 7.62%), maternal BMI at early pregnancy (*N* = 208,539, 14.12%), smoking during early pregnancy (*N* = 190,886, 7.37%) were not included in the corresponding analysis. Individuals with essential hypertension or pregestational diabetes were not included in the sibling analysis.

HRs and 95% CIs were estimated from multivariable Cox regression models.

*N* number, *IR* incidence rate (per 1000 person-years in crude), *HR* hazard ratio, *CI* confidence interval.

^a^HRs were stratified on calendar year of birth (1973–1977, 1978–1981, 1982–1986, 1987–1991, 1992–1996, 1997–2001, or 2002–2008), and adjusted for offspring sex (female or male), attained age, maternal country of birth (Nordic, non-Nordic, or unknown), maternal educational level (<10 years,10–11, 12, 13–14, 15+, or unknown), and history of parental psychiatric disorders.

^b^HRs were stratified on full-siblingship and adjusted for offspring sex (female or male), attained age, and calendar year of birth.

^c^This analysis was restricted to individuals born during 1992–2008 because information on maternal BMI at early pregnancy was largely complete from 1992 onward.

^d^This analysis was restricted to individuals born during 1982–2008 because information on maternal smoking during pregnancy was available from 1982 onward.

^e^This analysis was restricted to individuals born during 1987–2008 because information on maternal diabetes was available from 1987 onward.

### Perinatal factors

In the population analysis, we observed elevated risks of stress-related disorders among offspring born at 32–36 gestational weeks and birth weight for gestational age <10th percentile or >97th percentile. However, these associations were not observed in the sibling analyses. In population analysis, we found increased risk of stress-related disorders in individuals born at <32 gestational weeks (HR 1.35, 95% CI 1.22–1.49) and with a 5-minute Apgar score <7 (HR 1.22, 95% CI 1.12–1.32) (Table 3). In the sibling analysis, these HRs were attenuated to 1.16 (95% CI 0.93–1.45) and 1.13 (95% CI 0.95–1.33), respectively. Cesarean section was weakly associated with increased risk of stress-related disorders in both population (HR 1.09, 95% CI 1.06–1.12) and sibling (HR 1.10, 95% CI 1.02–1.20) analyses.

**Table 3:** Associations of perinatal factors with subsequent risk of stress-related disorders.

	Population analysis^a^			Sibling analysis^b^		
	Individuals, *N*	Cases, *N* (IR)	HR (95% CI)	Individuals, *N*	Cases, *N* (IR)	HR (95% CI)
Mode of delivery						
Unassisted vaginal delivery	2,807,264	46,403 (0.9)	1.00	2,129,565	31,700 (0.8)	1.00
Assisted vaginal delivery	223,453	3001 (0.8)	0.97 (0.94–1.01)	150,139	1880 (0.7)	1.03 (0.95–1.11)
Cesarean section	405,030	6107 (1.0)	1.09 (1.06–1.12)	274,531	3853 (0.9)	1.10 (1.02–1.20)
Gestational age, weeks						
<32	18,550	385 (1.3)	1.35 (1.22–1.49)	11,314	216 (1.2)	1.16 (0.93–1.45)
32–36	150,297	2816 (1.1)	1.15 (1.10–1.19)	103,199	1771 (1.0)	0.99 (0.91–1.07)
37–41	2,937,327	46,088 (0.9)	1.00	2,204,446	29,557 (0.8)	1.00
⩾42	329,573	6222 (0.9)	1.02 (1.00–1.05)	235,276	4034 (0.9)	1.00 (0.95–1.06)
Birth weight for gestational age, percentile						
<3rd	104,580	2565 (1.2)	1.23 (1.18–1.28)	67,113	1469 (1.1)	1.00 (0.91–1.10)
3rd to <10th	242,603	4948 (1.1)	1.10 (1.07–1.14)	165,389	3033 (0.9)	1.00 (0.94–1.06)
10th to 90th	2,765,814	43,273 (0.9)	1.00	2,068,852	29,557 (0.8)	1.00
>90th to 97th	197,625	2743 (0.8)	0.97 (0.93–1.00)	155,635	1985 (0.8)	0.98 (0.91–1.05)
>97th	112,165	1730 (0.9)	1.06 (1.01–1.11)	87,691	1224 (0.9)	1.06 (0.97–1.17)
Apgar score at 5 min^c^						
Low	31,572	608 (1.1)	1.22 (1.12–1.32)	21,139	363 (1.0)	1.13 (0.95–1.33)
Normal	3,182,721	49,080 (0.9)	1.00	2,392,981	33,762 (0.8)	1.00

Note: Individuals with missing information on birth weight for gestational age (*N* = 12,960, 0.38%) and missing information on 5 min Apgar score (*N* = 221,454, 6.45%) were not included in the corresponding analysis.

HRs and 95% CIs were estimated from multivariable Cox regression models.

*N* number, *IR* incidence rate (per 1000 person-years in crude), *HR* hazard ratio, *CI* confidence interval.

^a^HRs were stratified on calendar year of birth (1973–1977, 1978–1981, 1982–1986, 1987–1991, 1992–1996, 1997–2001, or 2002–2008), and adjusted for offspring sex (female or male), attained age, maternal country of birth (Nordic, non-Nordic, or unknown), maternal educational level (<10 years,10–11, 12, 13–14, 15+, or unknown), and history of parental psychiatric disorders.

^b^HRs were stratified on full-siblingship and adjusted for offspring sex (female or male), attained age, and calendar year of birth.

^c^Low Apgar score was defined as Apgar score < 7.

### Additional analyses

In the population analysis restricted to individuals with at least one sibling, the associations between parental and perinatal factors and risks of stress-related disorders were largely comparable to the associations noted in the full population cohort. However, a stronger association was noted between higher parity (i.e., the birth of fourth or higher order children) and stress-related disorders among individuals with siblings (Supplementary Table 3). We also obtained largely similar results for parental factors by mutually adjusting for parental factors in both population and sibling analyses (Supplementary Table 4), for perinatal factors after mutual adjustments (Supplementary Table 5) and after a finer adjustment for maternal country of birth (Supplementary Table 6). Largely comparable, yet weaker associations in population and sibling analyses were observed by restricting to stress-related disorders without psychiatric comorbidity, except for the increased risk among offspring of young fathers (paternal age at childbirth before 24 years) in the sibling analysis (Supplementary Tables 7, 8). Of note, the association between cesarean section delivery and stress-related disorders remained in the population analysis, but was further attenuated in the sibling analysis. In the analysis of subtypes of stress-related disorders, similar associations were observed between PTSD and ASR, except for moderately large differences in the associations between gestational age and stress-related disorders; the elevated risk among individuals born preterm in the population analysis remained in the sibling analysis for PTSD but not for ASR (Supplementary Table 9).

## Discussion

Using a nationwide birth cohort in Sweden with complete follow-up up to 35 years, our results do not support a causal role of many parental and perinatal factors in the development of stress-related disorders. The observed increased risks of stress-related disorders by adverse parental factors in the population analysis were largely attenuated to null in the sibling analysis, suggesting strong confounding by familial (environmental and genetic) factors shared by siblings. The associations of very preterm birth and low 5 min Apgar score with stress-related disorders were also attenuated by 40–60% in the sibling analysis, indicating that the association observed in the population analysis is largely driven by shared familial factors. The weak association we observed between cesarean section and risk of stress-related disorders in both population and sibling analyses was further attenuated in the sibling analysis when restricting to individuals without psychiatric comorbidities.

A growing body of evidence indicates that adverse fetal development (i.e., maternal diseases during pregnancy, low birth weight and preterm birth) may predispose individuals to mood and anxiety disorders [35], which are highly comorbid with stress-related disorders [36, 37]. However, none of these studies employed a sibling analysis to unmeasured confounding by familial environment and genetics [20]. The sibling analysis represents an advanced study design to account for confounders that are shared within families and fixed over time [38, 39]. In contrast, the population analysis inevitably incorporates a number of unmeasured confounders that are inherently controlled for when comparing siblings. For example, accumulating evidence suggests that familial factors, particularly genetic factors, drive the associations between maternal smoking during pregnancy and offspring cognitive and behavioral problems [20]. It is likely that familial factors also account for the increased risk of stress-related disorders in the population analysis since the association is attenuated to null in the sibling comparison. The sibling design thus has strong implications for causal inferences which is the purpose of the present study.

Our findings suggest that the increased risk of stress-related disorders among individuals exposed to parental adversity in the population setting is potentially explained by familial characteristics that are clustered within the family rather than putative biological effects. Other parental factors such as maternal smoking, overweight during pregnancy are highly correlated with familial environment. For instance, smoking and obesity are common in socially deprived households who are also at risk for PTSD [40–42]. Our data illustrate that the elevated risk of stress-related disorders among individuals with maternal smoking or overweight during pregnancy in the population analysis was attenuated to null when comparing within siblings, who share the familial environment in early life.

However, several perinatal adversities, e.g., preterm birth and small for gestational age, may to a large extent be attributable to genetic factors [43, 44]. Indeed, emerging evidence supports the contribution of the individual’s genetic makeup to stress-related disorders [45]. Twin studies have indicated that the heritability of PTSD ranges from 23.5 to 71% [46, 47], while recent genome-wide association studies have successfully identified robust genetic markers associated with PTSD and stress-related disorders [9, 48]. We found that the increased risk of stress-related disorders associated with a few perinatal factors in the population analysis were attenuated by 40–60% when comparing with full siblings, suggesting a genetic overlap between these factors and stress-related disorders. Preterm birth has been be associated with a range of psychiatric disorders, including bipolar affective disorder, autism spectrum disorders, substance use disorders, and attention‐deficit/hyperactivity disorder [19]. The heritability of preterm birth is estimated up to 40% in a twin study [49] and 20% in a genome-wide association study [50]. It is not implausible that the observed association between preterm birth and stress-related disorders is explained by genetic factors that may influence both outcomes. It has been reported that low 5 min Apgar score is associated with less stress resilience in young adulthood [51]. Although the Apgar score can be seen as a proxy to many parental and perinatal conditions, the heritability is estimated to be 42% [52]. Further research is needed to confirm the shared genetic contribution between 5 min Apgar scores and stress-related disorders.

Previous studies have indicated that cesarean section is associated with increased risks of attention deficit and hyperactivity disorder, tic disorders, and obsessive-compulsive disorder in both population and sibling analyses [17, 18]. Our data showed that individuals born by cesarean section are at mildly increased risk for stress-related disorders later in life. Although cesarean section is a result of many parental and intrapartum complications, this weak association remained after mutual adjustment for perinatal factors. However, the association was further attenuated by restricting to individuals without psychiatric comorbidities in the sibling analysis, suggesting that it may partly be driven by differences in the indication for cesarean section.

In line with previous studies [12], we observed higher IRs of stress-related disorder prevalence among female offsprings, offspring of mothers with lower educational attainment, and those not living with a partner, which are highly correlated with socioeconomic status [53, 54]. We also noted a lower IR of stress-related disorders among individuals born after 1997 which may be due to the fact that they had not reached the high-risk ages (median age at onset = 30 years, 25th percentile = 17 years) during the follow-up through 2013 [55]. The elevated IRs among offspring of parents with a history of psychiatric disorders before pregnancy may be driven by both genetic factors and familial environment [55].

To the best of our knowledge, this is the first study to comprehensively investigate parental and perinatal risk factors for stress-related disorders later in life. A major strength of our study was the large-scale nationwide birth cohort with long-term follow-up, and a sibling-based design. With prospectively and independently collected data on both exposures and outcome, our analysis was minimally affected by selection or information biases. The sibling analysis showed that most associations observed in the population analysis are strongly biased by unmeasured familial confounding, highlighting the importance of using sibling designs when studying associations of this kind in epidemiological research [56].

Our study also has limitations. First, we only included stress-related disorders diagnosed in hospital in-patient and out-patient care. Moreover, we lacked information on outpatient diagnoses before 2001. Although fairly good quality has been indicated for these usually specialist care-based diagnoses of stress-related disorders in the Swedish registers (the validity of PTSD diagnoses is >80%) [57], the present study does not capture stress-related disorders only diagnosed in primary care. However, individuals were always compared with their peers as we have stratified on birth year groups and used attained age at the underlying timescale in all analyses. Such underestimate of stress-related disorders is likely non-differential in relation to parental/perinatal factors and would have led to conservative results, partially responsible for the weaker estimates in sibling comparison. Second, because of the relatively young age of the individuals by the end of the follow-up, we only captured stress-related disorders up to age of 40 years. However, we covered the age band of high prevalence of stress-related disorders [12] and likely subtypes most relevant to early-life factors. Third, individuals with siblings might be somewhat different from individuals without siblings. However, we observed largely similar associations in the overall population analyses and when limiting the population analysis to those with at least one sibling. Furthermore, the population and sibling analysis does not account for confounding factors that are time-varying within-families [58] (e.g., non-shared environmental factors, birth order effect) [59], and may result in bias if there are carry-over effects among siblings (i.e., effects of the exposure and/or the outcome of one sibling on the exposure and/or outcome of subsequent siblings) [56]. Fourth, exposures that are likely constant across pregnancies cannot be addressed by the sibling analyses. For instance, we did not perform sibling analyses for pregestational diabetes and essential hypertension, because of very limited exposure variation between siblings. Moreover, mothers smoking during the first pregnancy are more likely to smoke during following pregnancies [60]. Although we included a simple classification on cigarette consumption (1–9 vs. ≥10 cigarettes per day), the full variation of maternal smoking across pregnancies might not be captured. Because time-varying parental/perinatal exposures do not occur at random and may be driven by other factors, we cannot rule out residual confounding. In addition, individuals exposed to parental or perinatal adversities are at risk for traumatic events in childhood or adolescence [61], leading to increased risk of stress-related disorder [62]. The mediating role traumatic events should be explored in future studies. Last, we only included a number of parental and perinatal factors that are common and well-documented in the Swedish MBR; less common exposures may also be of importance for the development of stress-related disorders and should be addressed in future studies.

In summary, our findings do not support an important role of many common parental or perinatal factors in the development of stress-related disorders later in life after careful consideration of familial confounders. Our study highlights the importance of taking familial confounders into account in observational studies of complex traits, such as stress-related disorders. Although these parental and perinatal factors are unlikely causally linked to stress-related disorders, the associations observed in the population analysis may guide strategies to identify high-risk groups.

## Supplementary information

supplemental material
