# Supplementary material for: Associations of parental and perinatal factors with subsequent risk of stress-related disorders: a nationwide cohort study with sibling comparison
Source: Mol Psychiatry. 2022 Jan 1;27(3):1712–9. doi: 10.1038/s41380-021-01406-5 (PMC9095463; doi:10.1038/s41380-021-01406-5)
Supplement: Supplementary file 1 — supplemental material [file 41380_2021_1406_MOESM1_ESM.docx]

**Supplementary Online Content**

**Supplementary Table 1.** International Classification of Diseases (ICD) codes for maternal and birth characteristics and stress-related disorders.

**Supplementary Table 2.** Numbers of families and siblings discordant on studied parental/perinatal factors and stress-related disorders

**Supplementary Table 3.** Associations of parental and perinatal factors with subsequent risk of stress-related disorders in the population analysis restricted to individuals with at least one sibling

**Supplementary Table 4.** Mutually-adjusted associations of parental factors with subsequent risk of stress-related disorders

**Supplementary Table 5.** Mutually-adjusted associations of perinatal factors with subsequent risk of stress-related disorders

**Supplementary Table 6.** Associations of parental and perinatal factors with subsequent risk of stress-related disorders, with finer adjustment for maternal country of birth in the population analysis

**Supplementary Table 7.** Associations of parental factors with subsequent risk of stress-related disorders without psychiatric comorbidity

**Supplementary Table 8.** Associations of perinatal factors with subsequent risk of stress-related disorders without psychiatric comorbidity

**Supplementary Table 9.** Associations of parental and perinatal factors with subsequent risks of post-traumatic disorder and acute stress disorder

**Supplementary Table 1.** International Classification of Diseases (ICD) codes for maternal and birth characteristics and stress-related disorders.

|  | **ICD-8 codes**  (1969-1986) | **ICD-9 codes**  (1987-1996) | **ICD-10 codes**  (1997-) |
| --- | --- | --- | --- |
| **Maternal and birth characteristics** |  |  |  |
| **Maternal hypertensive diseases** |  |  |  |
| Preeclampsia | 637.03-637.99 | 642E-642G | O14-O15 |
| Essential hypertension | 400-404 | 401-405, 642A-642C, 642H | O10, O11, I10-I15 |
| **Maternal diabetes** |  |  |  |
| Gestational diabetes | - | 648W | O244 |
| Pregestational diabetes | 250 | 250, 648A | O240-O243, E10-E14 |
| **Psychiatric disorders** | 290-319 | 290-319, 648E | F10-F99, O993 |
|  |  |  |  |
| **Stress-related disorders** | 307, 308.4 | 308, 309 | F43 |

**Supplementary Table 2.** Numbers of families and siblings discordant on studied parental/perinatal factors and stress-related disorders.

|  | **Number of distinct family with discordant exposure and outcome** | **Number of distinct siblings with discordant exposure and outcome** | **Number of outcome among siblings discordant exposure and outcome** |
| --- | --- | --- | --- |
| **Parental factors** |  |  |  |
| Maternal age at childbirth | 26,470 | 65,779 | 25,585 |
| Paternal age at childbirth | 24,576 | 35,037 | 14,504 |
| Parity | 34,296 | 88,498 | 35,251 |
| Maternal cohabitation status | 12,911 | 36,092 | 13,455 |
| Maternal BMI at early pregnancy, kg/m^2^ ^a^ | 7,207 | 11,586 | 3,394 |
| Maternal smoking during early pregnancy ^b^ | 7,649 | 20,480 | 7,403 |
| Preeclampsia | 1,577 | 4,408 | 1,641 |
| Gestational diabetes ^c^ | 310 | 710 | 237 |
| **Perinatal factors** |  |  |  |
| Mode of delivery | 6,852 | 18,501 | 7,078 |
| Gestational age, weeks | 10,629 | 29,813 | 11,056 |
| Birth weight for gestational age, percentile | 13,189 | 36,960 | 13,715 |
| Apgar score at 5 minutes ^d^ | 6,087 | 16,786 | 6,320 |

^a^ This analysis was restricted to individuals born during 1992-2008 because information on maternal BMI at early pregnancy was largely complete from 1992 onward.

^b^ This analysis was restricted to individuals born during 1982-2008 because information on smoking during pregnancy was available from 1982 onward.

^c^ This analysis was restricted to individuals born during 1987-2008 because information on maternal diabetes was available from 1987 onward.

^d^ Low Apgar score was defined as Apgar score <7.

**Supplementary Table 3.** Associations of parental and perinatal factors with subsequent risk of stress-related disorders in the population analysis restricted to individuals with at least one sibling.

|  | **Individuals, N** | **Case, N (IR)** | **HR (95% CI) ^a^** |
| --- | --- | --- | --- |
| **Maternal age at childbirth, years** |  |  |  |
| <20 | 65,365 | 2,234 (1.5) | 1.70 (1.62-1.78) |
| 20-24 | 550,006 | 11,439 (1.0) | 1.20 (1.17-1.23) |
| 25-29 | 955,914 | 13,415 (0.8) | 1.00 |
| 30-34 | 700,410 | 7,472 (0.7) | 0.97 (0.95-1.00) |
| ⩾35 | 282,540 | 2,873 (0.8) | 1.09 (1.04-1.13) |
| **Paternal age at childbirth, years** |  |  |  |
| <20 | 12,051 | 452 (1.7) | 1.84 (1.68-2.02) |
| 20-24 | 268,507 | 6,418 (1.1) | 1.26 (1.22-1.30) |
| 25-29 | 813,199 | 13,024 (0.8) | 1.00 |
| 30-34 | 835,673 | 10,270 (0.7) | 0.95 (0.93-0.98) |
| ⩾35 | 623,656 | 7,242 (0.8) | 1.06 (1.03-1.09) |
| **Maternal cohabitation status** |  |  |  |
| Non-cohabitation | 688,027 | 15,949 (0.9) | 0.97（0.93-1.02） |
| Cohabitation | 1,814,787 | 20,435 (0.8) | 1.00 |
| **Parity** |  |  |  |
| 1 | 958,719 | 14,326 (0.8) | 1.00 |
| 2 | 1,042,972 | 13,961 (0.8) | 1.02 (1.00-1.05) |
| 3 | 394,508 | 6,023 (0.9) | 1.13 (1.09-1.16) |
| ⩾4 | 158,036 | 3,123 (1.2) | 1.45 (1.39-1.51) |
| **Maternal BMI at early pregnancy, kg/m^2^ ^b^** |  |  |  |
| <18.5 | 25,176 | 160 (0.6) | 1.18 (1.01-1.39) |
| 18.5 to <25 | 650,967 | 2,962 (0.5) | 1.00 |
| 25 to <30 | 242,749 | 1,152 (0.5) | 1.17 (1.09-1.25) |
| ⩾30 | 95,155 | 501(0.7) | 1.45 (1.31-1.59) |
| **Maternal smoking during early pregnancy ^c^** |  |  |  |
| No smoking | 1,556,424 | 14,282 (0.7) | 1.00 |
| 1–9 cigarettes per day | 201,086 | 4,091 (1.2) | 1.46 (1.41-1.52) |
| ⩾10 cigarettes per day | 107,883 | 2,907 (1.5) | 1.75 (1.68-1.83) |
| **Maternal hypertensive diseases** |  |  |  |
| No | 2,476,248 | 36,412 (0.8) | 1.00 |
| Preeclampsia | 63,036 | 848 (0.8) | 0.98 (0.91-1.05) |
| Essential hypertension | 14,951 | 173 (1.0) | 1.23 (1.06-1.43) |
| **Maternal diabetes ^d^** |  |  |  |
| No | 1,617,891 | 14,241 (0.7) | 1.00 |
| Gestational diabetes | 13,742 | 127 (0.8) | 1.15 (0.97-1.37) |
| Pregestational diabetes | 9,784 | 102 (0.9) | 1.19 (0.98-1.45) |
| **Birth weight for gestational age, percentile** |  |  |  |
| <3^rd^ | 67,113 | 1,469 (1.1) | 1.22 (1.16-1.29) |
| 3^rd^ to <10^th^ | 165,389 | 3,033 (0.9) | 1.08 (1.04-1.13) |
| 10^th^ to 90^th^ | 2,068,852 | 29,557 (0.8) | 1.00 |
| >90^th^ to 97^th^ | 155,635 | 1,985 (0.8) | 0.99 (0.95-1.04) |
| >97^th^ | 87,691 | 1,224 (0.9) | 1.09 (1.03-1.16) |
| **Mode of delivery** |  |  |  |
| Unassisted vaginal delivery | 2,129,565 | 31,700 (0.8) | 1.00 |
| Assisted vaginal delivery | 150,139 | 1,880 (0.7) | 0.93 (0.89-0.98) |
| Cesarean section | 274,531 | 3,853 (0.9) | 1.11 (1.07-1.15) |
| **Gestational age, weeks** |  |  |  |
| <32 | 11,314 | 216 (1.2) | 1.39 (1.21-1.59) |
| 32-36 | 103,199 | 1,771 (1.0) | 1.17 (1.12-1.23) |
| 37-41 | 2,204,446 | 31,412 (0.8) | 1.00 |
| ⩾42 | 235,276 | 4,034 (0.9) | 1.02 (1.00-1.06) |
| **Apgar score at 5 minutes ^e^** |  |  |  |
| Low | 21,139 | 363 (1.0) | 1.00 |
| Normal | 2,392,981 | 33,762 (0.8) | 1.20 (1.08-1.33) |

Note: Individuals with missing information on paternal age at childbirth (N= 1,149, 0.45%), maternal cohabitation status (N=51,421, 2.01%), maternal BMI at early pregnancy (N=172,297, 14.52%), smoking during early pregnancy (N=146,021, 7.26%), birth weight for gestational age (N=9,555, 0.37%) and missing information on Apgar score at 5 minutes (N=140,115, 5.49 %) were not included in the corresponding analysis.

N, number; IR, incidence rate (per 1,000 person-years in crude); HR, hazard ratio; CI, confidence interval.

^a^ HRs were stratified on calendar year of birth (1973-1977, 1978-1981, 1982-1986, 1987-1991, 1992-1996, 1997-2001, or 2002-2008), and adjusted for offspring sex (female or male), attained age, maternal country of birth (Nordic, non-Nordic, or unknown), maternal educational level (<10 years,10-11, 12, 13-14, 15+, or unknown), and history of parental psychiatric disorders.

^b^ This analysis was restricted to individuals born during 1992-2008 because information on maternal BMI at early pregnancy was largely complete from 1992 onward.

^c^ This analysis was restricted to individuals born during 1982-2008 because information on maternal smoking during pregnancy was available from 1982 onward.

^d^ This analysis was restricted to individuals born during 1987-2008 because information on maternal diabetes was available from 1987 onward.

^e^ Low Apgar score was defined as Apgar score <7.

**Supplementary Table 4.** Mutually-adjusted associations of parental factors with subsequent risk of stress-related disorders.

|  | **Population analysis ^a^** | **Sibling analysis ^b^** |
| --- | --- | --- |
|  | **HR (95% CI)** | **HR (95% CI)** |
| **Maternal age at childbirth, years** |  |  |
| <20 | 1.59(1.52-1.66) | 1.00(0.89-1.12) |
| 20-24 | 1.21(1.18-1.24) | 0.97(0.92-1.03) |
| 25-29 | 1.00 | 1.00 |
| 30-34 | 0.91 (0.89-0.93) | 1.01(0.95-1.08) |
| ⩾35 | 0.91(0.88-0.94) | 1.00(0.88-1.13) |
| **Paternal age at childbirth, years** |  |  |
| <20 | 1.37(1.29-1.48) | 1.13(0.93-1.36) |
| 20-24 | 1.14(1.11-1.17) | 1.04(0.97-1.11) |
| 25-29 | 1.00 | 1.00 |
| 30-34 | 0.98(0.96-1.00) | 0.99(0.93-1.05) |
| ⩾35 | 1.05(1.02-1.08) | 0.97(0.88-1.08) |
| **Maternal cohabitation status** |  |  |
| Non-cohabitation | 1.20(1.17-1.23) | 0.99(0.93-1.05) |
| Cohabitation | 1.00 | 1.00 |
| **Parity** |  |  |
| 1 | 1.00 | 1.00 |
| 2 | 1.08(1.06-1.11) | 0.93(0.89-0.98) |
| 3 | 1.25(1.23-1.30) | 0.89(0.82-0.96) |
| ⩾4 | 1.56(1.51-1.62) | 0.82(0.72-0.93) |
| **Maternal BMI at early pregnancy, kg/m^2^ ^c^** |  |  |
| <18.5 | 0.99(0.86-1.14) | 0.88(0.65-1.18) |
| 18.5 to <25 | 1.00 | 1.00 |
| 25 to <30 | 1.13(1.06-1.19) | 1.06(0.93-1.21) |
| ⩾30 | 1.32(1.22-1.43) | 1.05(0.87-1.27) |
| **Maternal smoking during early pregnancy ^d^** |  |  |
| No smoking | 1.00 | 1.00 |
| 1–9 cigarettes per day | 1.40(1.36-1.44) | 1.03(0.95-1.11) |
| ⩾10 cigarettes per day | 1.58(1.52-1.64) | 0.99(0.90-1.08) |
| **Maternal hypertensive diseases** |  |  |
| No | 1.00 | 1.00 |
| Preeclampsia | 1.04(0.99-1.05) | 1.04(0.92-1.17) |
| Essential hypertension | 1.20(1.05-1.35) | - |
| **Maternal diabetes ^e^** |  |  |
| No | 1.00 | 1.00 |
| Gestational diabetes | 1.14(0.98-1.32) | 0.82(0.61-1.10) |
| Pregestational diabetes | 1.17(1.00-1.38) | - |

Note: Individuals with missing information on paternal age at childbirth (N=23,720, 0.69%), maternal cohabitation status (N=261,739, 7.62%), maternal BMI at early pregnancy (N=208,539, 14.12%), smoking during early pregnancy (N=190,886, 7.37%) were not included in the corresponding analysis. Individuals with essential hypertensions or pregestational diabetes were not included in the sibling analysis

HR, hazard ratio; CI, confidence interval.

^a^ HRs were stratified on calendar year of birth (1973-1977, 1978-1981, 1982-1986, 1987-1991, 1992-1996, 1997-2001, or 2002-2008), and adjusted for offspring sex (female or male), attained age, maternal country of birth (Nordic, non-Nordic, or unknown), maternal educational level (<10 years, 10-11, 12, 13-14, 15+, or unknown), and mutually adjusted for factors presented in this table.

^b^ HRs were stratified on full-siblingship and adjusted for offspring sex (female or male), attained age, calendar year of birth, history of parental psychiatric disorders, and mutually adjusted for factors presented in this table.

^c^ This analysis was restricted to individuals born during 1992-2008 because information on maternal BMI at early pregnancy was largely complete from 1992 onward.

^d^ Because information on smoking during pregnancy was available from 1982 onward, this analysis was restricted to individuals born during 1982-2008.

^e^ This analysis was restricted to individuals born during 1987-2008 because information on maternal diabetes was available from 1987 onward.

**Supplementary Table 5.** Mutually-adjusted associations of perinatal factors with subsequent risk of stress-related disorders.

|  | **Population analysis ^a^** | **Sibling analysis ^b^** |
| --- | --- | --- |
|  | **HR (95% CI)** | **HR (95% CI)** |
| **Birth weight for gestational age, percentile** |  |  |
| <3^rd^ | 1.20(1.15-1.25) | 0.98(0.90-1.08) |
| 3^rd^ to <10^th^ | 1.10(1.07-1.13) | 0.99(0.93-1.05) |
| 10^th^ to 90^th^ | 1.00 | 1.00 |
| >90^th^ to 97^th^ | 0.96(0.93-1.00) | 0.99(0.92-1.06) |
| >97^th^ | 1.04(1.00-1.09) | 1.08(0.98-1.19) |
| **Mode of delivery** |  |  |
| Unassisted vaginal delivery | 1.00 | 1.00 |
| Assisted vaginal delivery | 0.97(0.93-1.00) | 1.00(0.93-1.09) |
| Cesarean section | 1.06(1.03-1.09) | 1.10(1.02-1.19) |
| **Gestational age, weeks** |  |  |
| <32 | 1.25(1.13-1.25) | 1.11(0.88-1.39) |
| 32-36 | 1.11(1.07-1.16) | 0.97(0.89-1.05) |
| 37-41 | 1.00 | 1.00 |
| ⩾42 | 1.01(0.98-1.03) | 1.00 (0.95-1.06) |
| **Apgar score at 5 minutes ^c^** |  |  |
| Low | 1.14(1.05-1.23) | 1.13 (0.96-1.34) |
| Normal | 1.00 | 1.00 |

Note: Individuals with missing information on birth weight for gestational age (N=12,960, 0.38%) and missing information on Apgar score at 5 and 10 minutes (N=204,520, 5.95 %) were not included in the corresponding analysis.

HR, hazard ratio; CI, confidence interval.

^a^ HRs were stratified on calendar year of birth (1973-1977, 1978-1981, 1982-1986, 1987-1991, 1992-1996, 1997-2001, or 2002-2008), and adjusted for offspring sex (female or male), attained age, maternal country of birth (Nordic, non-Nordic, or unknown), maternal educational level (<10 years, 10-11, 12, 13-14, 15+, or unknown), and mutually adjusted for factors presented in this table.

^b^ HRs were stratified on full-siblingship and adjusted for offspring sex (female or male), attained age, calendar year of birth, history of parental psychiatric disorders, and mutually adjusted for factors presented in this table.

^c^ Low Apgar score was defined as Apgar score <7.

**Supplementary Table 6.** Associations of parental and perinatal factors with subsequent risk of stress-related disorders, with finer adjustment for maternal country of birth in the population analysis

|  | **Population analysis ^a^** |
| --- | --- |
|  | **HR (95% CI)** |
| **Maternal age at childbirth, years** |  |
| <20 | 1.84 (1.78-1.91) |
| 20-24 | 1.25 (1.22-1.27) |
| 25-29 | 1.00 |
| 30-34 | 0.97 (0.95-1.00) |
| ⩾35 | 1.09 (1.06-1.13) |
| **Paternal age at childbirth, years** |  |
| <20 | 1.96 (1.84-2.08) |
| 20-24 | 1.32 (1.29-1.35) |
| 25-29 | 1.00 |
| 30-34 | 0.93 (0.91-0.95) |
| ⩾35 | 1.03 (1.01-1.06) |
| **Maternal cohabitation status** |  |
| Non-cohabitation | 1.01 (0.97-1.05) |
| Cohabitation | 1.00 |
| **Parity** |  |
| 1 | 1.00 |
| 2 | 0.91 (0.89-0.93) |
| 3 | 0.99 (0.97-1.01) |
| ⩾4 | 1.25 (1.21-1.29) |
| **Maternal BMI at early pregnancy, kg/m^2^ ^b^** |  |
| <18.5 | 1.09 (0.95-1.26) |
| 18.5 to <25 | 1.00 |
| 25 to <30 | 1.14 (1.07-1.21) |
| ⩾30 | 1.38 (1.27-1.49) |
| **Maternal smoking during early pregnancy ^c^** |  |
| No smoking | 1.00 |
| 1–9 cigarettes per day | 1.51 (1.47-1.55) |
| ⩾10 cigarettes per day | 1.79 (1.74-1.86) |
| **Maternal hypertensive diseases** |  |
| No | 1.00 |
| Preeclampsia | 1.02 (0.96-1.07) |
| Essential hypertension | 1.17 (1.03-1.33) |
| **Maternal diabetes ^d^** |  |
| No | 1.00 |
| Gestational diabetes | 1.16 (1.00-1.34) |
| Pregestational diabetes | 1.22 (1.04-1.44) |
| **Birth weight for gestational age, percentile** |  |
| <3^rd^ | 1.26 (1.21-1.31) |
| 3^rd^ to <10^th^ | 1.12 (1.09-1.15) |
| 10^th^ to 90^th^ | 1.00 |
| >90^th^ to 97^th^ | 0.96 (0.92-1.00) |
| >97^th^ | 1.06 (1.01-1.11) |
| **Mode of delivery** |  |
| Unassisted vaginal delivery | 1.00 |
| Assisted vaginal delivery | 0.97(0.94-1.01) |
| Cesarean section | 1.11(1.08-1.14) |
| **Gestational age, weeks** |  |
| <32 | 1.40 (1.26-1.54) |
| 32-36 | 1.17 (1.13-1.22) |
| 37-41 | 1.00 |
| ⩾42 | 1.03 (1.00-1.05) |
| **Apgar score at 5 minutes ^e^** |  |
| Low | 1.24 (1.14-1.34) |
| Normal | 1.00 |

Note: Individuals with missing information on paternal age at childbirth (N=23,720, 0.69%), maternal cohabitation status (N=261,739, 7.62%), maternal BMI at early pregnancy (N=208,539, 14.12%), smoking during early pregnancy (N=190,886, 7.37%) were not included in the corresponding analysis. Individuals with essential hypertension or pregestational diabetes were not included in the sibling analysis. Individuals with missing information on birth weight for gestational age (N=12,960, 0.38%) and missing information on Apgar score at 5 and 10 minutes (N=204,520, 5.95 %) were not included in the corresponding analysis.

HR, hazard ratio; CI, confidence interval.

^a^ HRs were stratified on calendar year of birth (1973-1977, 1978-1981, 1982-1986, 1987-1991, 1992-1996, 1997-2001, or 2002-2008), and adjusted for offspring sex (female or male), attained age, maternal country of birth (Sweden, other Nordic, Europe, Africa, Asia, other countries), maternal educational level (<10 years,10-11, 12, 13-14, 15+, or unknown), and history of parental psychiatric disorders.

^b^ This analysis was restricted to individuals born during 1992-2008 because information on maternal BMI at early pregnancy was largely complete from 1992 onward.

^c^ Because information on smoking during pregnancy was available from 1982 onward, this analysis was restricted to individuals born during 1982-2008.

^d^ This analysis was restricted to individuals born during 1987-2008 because information on maternal diabetes was available from 1987 onward.

^e^ Low Apgar score was defined as Apgar score <7.

**Supplementary Table 7.** Associations of parental factors with subsequent risk of stress-related disorders without psychiatric comorbidity

|  | **Population analysis ^a^** | | **Sibling analysis ^b^** | |
| --- | --- | --- | --- | --- |
|  | Cases, N (IR) | HR (95% CI) | Cases, N (IR) | HR (95% CI) |
| **Maternal age at childbirth, years** |  |  |  |  |
| <20 | 1,314(0.5) | 1.58(1.49-1.68) | 773(0.5) | 1.17(0.96-1.42) |
| 20-24 | 5,481(0.4) | 1.17(1.13-1.21) | 3,981(0.3) | 1.00(0.90-1.11) |
| 25-29 | 6,606(0.3) | 1.00 | 4,807(0.3) | 1.00 |
| 30-34 | 3,961(0.3) | 0.96(0.93-1.00) | 2,654(0.3) | 1.02(0.92-1.14) |
| ⩾35 | 1,758(0.3) | 1.01(0.96-1.07) | 938(0.3) | 0.95(0.77-1.17) |
| **Paternal age at childbirth, years** |  |  |  |  |
| <20 | 291(0.6) | 1.61(1.43-1.81) | 145(0.5) | 1.49(1.10-2.03) |
| 20-24 | 3,280(0.4) | 1.24(1.19-1.29) | 2,268(0.4) | 1.11(1.00-1.24) |
| 25-29 | 6,350(0.3) | 1.00 | 4,670(0.3) | 1.00 |
| 30-34 | 5,115(0.3) | 0.94(0.90-0.97) | 3,651(0.3) | 0.96(0.87-1.06) |
| ⩾35 | 3,921(0.3) | 0.99(0.95-1.03) | 2,415(0.3) | 0.92(0.77-1.09) |
| **Maternal cohabitation status** |  |  |  |  |
| Non-cohabitation | 3,944(0.4) | 1.22(1.18-1.27) | 2,154(0.4) | 1.03(0.93-1.13) |
| Cohabitation | 13,397(0.3) | 1.00 | 9,780 (0.3) | 1.00 |
| **Parity** |  |  |  |  |
| 1 | 8,062(0.3) | 1.00 | 5,232(0.3) | 1.00 |
| 2 | 6,687(0.3) | 0.93(0.90-0.96) | 4,919(0.3) | 0.98(0.91-1.05) |
| 3 | 2,925(0.3) | 0.97(0.93-1.02) | 1,985(0.3) | 0.94(0.82-1.08) |
| ⩾4 | 1,446(0.4) | 1.19(1.13-1.26) | 1,017(0.4) | 0.95(0.77-1.19) |
| **Maternal cohabitation status** |  |  |  |  |
| Non-cohabitation | 3,944(0.4) | 1.22(1.18-1.27) | 2,154(0.4) | 1.03(0.93-1.13) |
| Cohabitation | 13,397(0.3) | 1.00 | 9,780 (0.3) | 1.00 |
| **Maternal BMI at early pregnancy, kg/m^2^ ^c^** |  |  |  |  |
| <18.5 | 73(0.2) | 0.99(0.79-1.26) | 55(0.2) | 0.53(0.22-1.31) |
| 18.5 to <25 | 1,503(0.2) | 1.00 | 1,112(0.2) | 1.00 |
| 25 to <30 | 590(0.2) | 1.14(1.04-1.25) | 453(0.2) | 1.26(0.84-1.89) |
| ⩾30 | 246(0.3) | 1.29(1.13-1.48) | 162(0.2) | 1.05(0.53-2.10) |
| **Maternal smoking during early pregnancy ^d^** |  |  |  |  |
| No smoking | 6,153(0.3) | 1.00 | 4,971(0.2) | 1.00 |
| 1–9 cigarettes per day | 1,853(0.4) | 1.34(1.27-1.42) | 1,295(0.4) | 0.98(0.83-1.14) |
| ⩾10 cigarettes per day | 1,318(0.5) | 1.48(1.39-1.57) | 862(0.5) | 1.00(0.82-1.22) |
| **Maternal hypertensive diseases** |  |  |  |  |
| No | 18,590(0.3) | 1.00 | 12,815(0.3) | 1.00 |
| Preeclampsia | 454(0.3) | 0.98(0.89-1.08) | 283(0.3) | 0.97(0.79-1.2) |
| Essential hypertension | 76(0.3) | 1.08(0.86-1.35) | - | - |
| **Maternal diabetes ^e^** |  |  |  |  |
| No | 6,370(0.3) | 1.00 | 4,823(0.3) | 1.00 |
| Gestational diabetes | 64(0.3) | 1.18(0.92-1.51) | 45(0.3) | 0.82(0.43-1.59) |
| Pregestational diabetes | 49(0.3) | 1.19(0.90-1.57) | - | - |

Note: Individuals with missing information on paternal age at childbirth (N=23,720, 0.69%), maternal cohabitation status (N=261,739, 7.62%), maternal BMI at early pregnancy (N=208,539, 14.12%), smoking during early pregnancy (N=190,886, 7.37%), birth weight for gestational age (N=12,960, 0.38%) were not included in the corresponding analysis. Individuals with essential hypertensions or pregestational diabetes were not included in the sibling analysis.

N, number; IR, incidence rate (per 1,000 person-years in crude); HR, hazard ratio; CI, confidence interval.

^a^ HRs were stratified on calendar year of birth (1973-1977, 1978-1981, 1982-1986, 1987-1991, 1992-1996, 1997-2001, or 2002-2008), and adjusted for offspring sex (female or male), attained age, maternal country of birth (Nordic, non-Nordic, or unknown), maternal educational level (<10 years, 10-11, 12, 13-14, 15+, or unknown), and history of parental psychiatric disorders.

^b^ HRs were stratified on full-siblingship and adjusted for offspring sex (female or male), attained age, calendar year of birth.

^c^ This analysis was restricted to individuals born during 1992-2008 because information on maternal BMI at early pregnancy was largely complete from 1992 onward.

^d^ This analysis was restricted to individuals born during 1982-2008 because information on smoking during pregnancy was available from 1982 onward.

^e^ This analysis was restricted to individuals born during 1987-2008 because information on maternal diabetes was available from 1987 onward.

**Supplementary Table 8.** Associations of perinatal factors with subsequent risk of stress-related disorders without psychiatric comorbidity.

|  | **Population analysis ^a^** | | **Sibling analysis ^b^** | |
| --- | --- | --- | --- | --- |
|  | Cases, N (IR) | HR (95% CI) | Cases, N (IR) | HR (95% CI) |
| **Birth weight for gestational age, percentile** |  |  |  |  |
| <3^rd^ | 868(0.4) | 1.20(1.12-1.29) | 510(0.4) | 0.88(0.76-1.03) |
| 3^rd^ to <10^th^ | 1,649(0.4) | 1.06(1.01-1.12) | 1,034(0.3) | 0.98(0.88-1.09) |
| 10^th^ to 90^th^ | 15,004(0.3) | 1.00 | 10,486(0.3) | 1.00 |
| >90^th^ to 97^th^ | 935(0.3) | 0.95(0.89-1.02) | 671(0.3) | 1.01(0.89-1.14) |
| >97^th^ | 589(0.3) | 1.04(0.96-1.13) | 408(0.3) | 1.06(0.89-1.25) |
| **Mode of delivery** |  |  |  |  |
| Unassisted vaginal delivery | 16,032(0.3) | 1.00 | 11,156(0.3) | 1.00 |
| Assisted vaginal delivery | 1,032(0.3) | 0.96(0.90-1.02) | 654(0.3) | 0.93(0.81-1.07) |
| Cesarean section | 2,056(0.3) | 1.08(1.03-1.13) | 1,343(0.3) | 1.04(0.91-1.20) |
| **Gestational age, weeks** |  |  |  |  |
| <32 | 116(0.4) | 1.21(1.00-1.45) | 71(0.4) | 1.33(0.89-1.99) |
| 32-36 | 889(0.3) | 1.06(0.99-1.13) | 583(0.3) | 0.88(0.77-1.01) |
| 37-41 | 15,910(0.3) | 1.00 | 11,004(0.3) | 1.00 |
| ⩾42 | 2,205(0.3) | 1.03(0.99-1.08) | 1,495(0.3) | 1.02(0.93-1.12) |
| **Apgar score at 5 minutes ^c^** |  |  |  |  |
| Low | 207(0.4) | 1.20(1.04-1.37) | 126(0.3) | 1.26(0.95-1.70) |
| Normal | 16,800(0.3) | 1.00 | 11,787(0.3) | 1.00 |

Note: Individuals with missing information on birth weight for gestational age (N=12,960, 0.38%) and missing information on Apgar score at 5 and 10 minutes (N=204,520, 5.95 %) were not included in the corresponding analysis.

N, number; IR, incidence rate (per 1,000 person-years in crude); HR, hazard ratio; CI, confidence interval.

^a^ HRs were stratified on calendar year of birth (1973-1977, 1978-1981, 1982-1986, 1987-1991, 1992-1996, 1997-2001, or 2002-2008), and adjusted for offspring sex (female or male), attained age, maternal country of birth (Nordic, non-Nordic, or unknown), maternal educational level (<10 years, 10-11, 12, 13-14, 15+, or unknown), and history of parental psychiatric disorders.

^b^ HRs were stratified on full-siblingship and adjusted for offspring sex (female or male), attained age, calendar year of birth.

^c^ Low Apgar score was defined as Apgar score <7.

**Supplementary Table 9. Associations of parental and perinatal factors with subsequent risks of post-traumatic disorder and acute stress disorder**

|  | **Post-traumatic stress disorder** | | | | **Acute stress disorder** | | | |
| --- | --- | --- | --- | --- | --- | --- | --- | --- |
|  | **Population analysis ^a^** | | **Sibling analysis ^b^** | | **Population analysis ^a^** | | **Sibling analysis ^b^** | |
|  | **Case, N (IR)** | **HR (95% CI)** | **Case, N (IR)** | **HR (95% CI)** | **Case, N (IR)** | **HR (95% CI)** | **Case, N (IR)** | **HR (95% CI)** |
| **Maternal age at childbirth, years** | | | | | | | | |
| <20 | 478 (0.2) | 1.97 (1.78-2.18) | 254 (0.2) | 0.84 (0.58-1.21) | 2,132 (0.8) | 1.85 (1.77-1.94) | 1,145 (0.7) | 0.98 (0.83-1.14) |
| 20-24 | 1,920 (0.1) | 1.31 (1.23-1.40) | 1,366 (0.1) | 1.03 (0.85-1.25) | 8,316 (0.6) | 1.26 (1.22-1.29) | 5,772 (0.5) | 0.93 (0.85-1.01) |
| 25-29 | 2,128 (0.1) | 1.00 | 1,574 (0.1) | 1.00 | 9,211 (0.4) | 1.00 | 6,692 (0.4) | 1.00 |
| 30-34 | 1,331 (0.1) | 0.97 (0.91-1.04) | 904 (0.1) | 1.07 (0.86-1.32) | 5,466 (0.4) | 0.97 (0.94-1.00) | 3,658 (0.3) | 1.03 (0.94-1.13) |
| ⩾35 | 613 (0.1) | 1.03 (0.94-1.12) | 360 (0.1) | 1.01 (0.67-1.50) | 2,592 (0.4) | 1.09 (1.05-1.14) | 1,399 (0.4) | 1.01 (0.84-1.20) |
| **Paternal age at childbirth, years** | | | | | | | | |
| <20 | 117 (0.2) | 2.06 (1.71-2.48) | 58 (0.2) | 0.92 (0.52-1.63) | 474 (0.9) | 1.86 (1.70-2.04) | 208 (0.8) | 0.98 (0.75-1.27) |
| 20-24 | 1,099 (0.1) | 1.30 (1.21-1.40) | 694 (0.1) | 0.89 (0.73-1.09) | 5,068 (0.6) | 1.34 (1.29-1.39) | 3,284 (0.6) | 1.04 (0.95-1.13) |
| 25-29 | 2,071 (0.1) | 1.00 | 1,528 (0.1) | 1.00 | 8,989 (0.4) | 1.00 | 6,485 (0.4) | 1.00 |
| 30-34 | 1,694 (0.1) | 0.92 (0.86-0.98) | 1,238 (0.1) | 0.95 (0.79-1.14) | 7,200 (0.4) | 0.94 (0.91-0.97) | 5,128 (0.4) | 0.98 (0.90-1.06) |
| ⩾35 | 1,406 (0.1) | 1.01 (0.94-1.08) | 934 (0.1) | 0.95 (0.68-1.33) | 5,690 (0.4) | 1.04 (1.00-1.07) | 3,546 (0.4) | 0.91 (0.79-1.06) |
| **Maternal cohabitation status** | | | | | | | | |
| Non-cohabitation | 424 (0.3) | 2.01 (1.82-2.22) | 174 (0.2) | 1.18 (0.87-1.60) | 1,368 (0.8) | 1.81 (1.71-1.91) | 460 (0.6) | 0.90 (0.76-1.06) |
| Cohabitation | 3,529 (0.1) | 1.00 | 2,762 (0.1) | 1.00 | 12,779 (0.4) | 1.00 | 10,079 (0.4) | 1.00 |
| **Parity** | | | | | | | | |
| 1 | 2,632 (0.1) | 1.00 | 1,587 (0.1) | 1.00 | 11,519 (0.5) | 1.00 | 6,987 (0.4) | 1.00 |
| 2 | 2,199 (0.1) | 0.93 (0.88-0.99) | 1,659 (0.1) | 1.01 (0.88-1.15) | 9,540 (0.4) | 0.92 (0.90-0.95) | 7,061 (0.4) | 0.99 (0.94-1.05) |
| 3 | 1,057 (0.1) | 1.04 (0.97-1.12) | 771 (0.1) | 1.03 (0.81-1.32) | 4,392 (0.5) | 1.00 (0.97-1.04) | 3,033 (0.5) | 0.97 (0.87-1.09) |
| ⩾4 | 582 (0.2) | 1.37 (1.25-1.50) | 441 (0.2) | 1.12 (0.76-1.64) | 2,266 (0.7) | 1.29 (1.24-1.35) | 1,585 (0.6) | 0.93 (0.77-1.11) |
| **Maternal BMI at early pregnancy, kg/m^2^ ^c^** | | | | | | | | |
| <18.5 | 42 (0.1) | 1.22 (0.90-1.67) | 34 (0.1) | 0.95 (0.27-3.37) | 80 (0.3) | 1.09 (0.87-1.37) | 61 (0.2) | 1.09 (0.49-2.45) |
| 18.5 to <25 | 703 (0.1) | 1.00 | 488 (0.1) | 1.00 | 1,520 (0.2) | 1.00 | 1,112 (0.2) | 1.00 |
| 25 to <30 | 283 (0.1) | 1.18 (1.03-1.35) | 204 (0.1) | 0.75 (0.41-1.39) | 555 (0.2) | 1.11 (1.01-1.23) | 407 (0.2) | 1.19 (0.76-1.87) |
| ⩾30 | 134 (0.1) | 1.55 (1.29-1.87) | 98 (0.1) | 0.37 (0.13-1.03) | 262 (0.3) | 1.51 (1.32-1.72) | 199 (0.3) | 1.08 (0.53-2.19) |
| **Maternal smoking during early pregnancy** | | | | | | | | |
| No smoking | 2,488 (0.1) | 1.00 | 1,973 (0.1) | 1.00 | 8,595 (0.4) | 1.00 | 6,860 (0.3) | 1.00 |
| 1–9 cigarettes per day | 789 (0.2) | 1.48 (1.36-1.61) | 516 (0.2) | 1.00 (0.76-1.31) | 3,104 (0.7) | 1.53 (1.47-1.60) | 2,120 (0.6) | 1.02 (0.90-1.16) |
| ⩾10 cigarettes per day | 644 (0.2) | 1.91 (1.75-2.09) | 412 (0.2) | 1.08 (0.79-1.49) | 2,293 (0.8) | 1.76 (1.68-1.85) | 1,441 (0.8) | 0.99 (0.84-1.16) |
| **Maternal hypertensive diseases** | | | | | | | | |
| No | 6,283 (0.1) | 1.00 | 4,338 (0.1) | 1.00 | 26,936 (0.5) | 1.00 | 18,179 (0.4) | 1.00 |
| Preeclampsia | 160 (0.1) | 0.99 (0.85-1.16) | 106 (0.1) | 1.26 (0.86-1.84) | 678 (0.5) | 1.01 (0.93-1.09) | 406 (0.4) | 1.01 (0.86-1.20) |
| Essential hypertension | 27 (0.1) | 0.92 (0.63-1.35) | - | - | 103 (0.4) | 1.03 (0.85-1.25) | - | - |
| **Maternal diabetes ^d^** | | | | | | | | |
| No | 2,961 (0.1) | 1.00 | 2,166 (0.1) | 1.00 | 8,827 (0.4) | 1.00 | 6,540 (0.3) | 1.00 |
| Gestational diabetes | 26 (0.1) | 1.04 (0.70-1.53) | 18 (0.1) | 0.51 (0.20-1.33) | 87 (0.4) | 1.22 (0.99-1.51) | 59 (0.4) | 0.72 (0.40-1.26) |
| Pregestational diabetes | 22 (0.2) | 1.18 (0.78-1.79) | - | - | 63 (0.4) | 1.11 (0.86-1.42) | - | - |
| **Birth weight for gestational age, percentile** | | | | | | | | |
| <3rd | 295 (0.1) | 1.30 (1.16-1.47) | 175 (0.1) | 1.11 (0.82-1.50) | 1,295 (0.6) | 1.25 (1.19-1.33) | 733 (0.5) | 0.97 (0.85-1.10) |
| 3rd to <10th | 563 (0.1) | 1.13 (1.03-1.23) | 348 (0.1) | 0.99 (0.81-1.22) | 2,471 (0.5) | 1.11 (1.06-1.16) | 1,520 (0.5) | 0.98 (0.90-1.08) |
| 10th to 90th | 5,039 (0.1) | 1.00 | 3,529 (0.1) | 1.00 | 21,618 (0.5) | 1.00 | 14,729 (0.4) | 1.00 |
| >90th to 97th | 329 (0.1) | 0.98 (0.87-1.09) | 226 (0.1) | 0.71 (0.56-0.90) | 1,358 (0.4) | 0.96 (0.91-1.01) | 1,010 (0.4) | 1.05 (0.95-1.17) |
| >97th | 218 (0.1) | 1.14 (0.99-1.30) | 162 (0.1) | 0.77 (0.57-1.04) | 857 (0.5) | 1.06 (0.99-1.13) | 594 (0.4) | 1.08 (0.94-1.24) |
| **Mode of delivery** | | | | | | | | |
| Unassisted vaginal delivery | 5,401 (0.1) | 1.00 | 3,790 (0.1) | 1.00 | 23,218 (0.5) | 1.00 | 15,791 (0.4) | 1.00 |
| Assisted vaginal delivery | 332 (0.1) | 0.95 (0.85-1.06) | 206 (0.1) | 1.03 (0.80-1.34) | 1,489 (0.4) | 0.96 (0.91-1.02) | 947 (0.4) | 1.03 (0.92-1.15) |
| Cesarean section | 737 (0.1) | 1.13 (1.04-1.22) | 462 (0.1) | 1.07 (0.82-1.40) | 3,010 (0.5) | 1.10 (1.06-1.14) | 1,928 (0.4) | 1.03 (0.92-1.15) |
| **Gestational age, weeks** | | | | | | | | |
| <32 | 48 (0.2) | 1.44 (1.08-1.91) | 27 (0.1) | 1.56 (0.79-3.09) | 187 (0.6) | 1.36 (1.18-1.57) | 104 (0.6) | 1.04 (0.76-1.42) |
| 32-36 | 348 (0.1) | 1.25 (1.12-1.39) | 217 (0.1) | 1.16 (0.90-1.51) | 1,422 (0.5) | 1.17 (1.11-1.24) | 878 (0.5) | 0.93 (0.83-1.04) |
| 37-41 | 5,400 (0.1) | 1.00 | 3,764 (0.1) | 1.00 | 22,966 (0.5) | 1.00 | 15,646 (0.4) | 1.00 |
| ⩾42 | 674 (0.1) | 1.03 (0.95-1.11) | 450 (0.1) | 1.10 (0.92-1.32) | 3,142 (0.5) | 1.03 (0.99-1.06) | 2,038 (0.4) | 1.01 (0.93-1.09) |
| **Apgar score at 5 minutes^e^** | | | | | | | | |
| Low | 63 (0.1) | 1.14 (0.89-1.47) | 39 (0.1) | 0.80 (0.46-1.39) | 311 (0.6) | 1.25 (1.12-1.40) | 187 (0.5) | 1.18 (0.93-1.50) |
| Normal | 5,851 (0.1) | 1.00 | 4,099 (0.1) | 1.00 | 24,580 (0.5) | 1.00 | 16,845 (0.4) | 1.00 |

Note: Analysis were restricted to follow-up from 1987 onward when PTSD and ASR diagnoses were distinguishable in registers.

N, number; IR, incidence rate (per 1,000 person-years in crude); HR, hazard ratio; CI, confidence interval.

^a^ HRs were stratified on calendar year of birth (1973-1977, 1978-1981, 1982-1986, 1987-1991, 1992-1996, 1997-2001, or 2002-2008), and adjusted for offspring sex (female or male), attained age, maternal country of birth (Nordic, non-Nordic, or unknown), maternal educational level (<10 years,10-11, 12, 13-14, 15+, or unknown), and history of parental psychiatric disorders.

^b^ HRs were stratified on full-siblingship and adjusted for offspring sex (female or male), attained age, and calendar year of birth.

^c^ This analysis was restricted to individuals born during 1992-2008 because information on maternal BMI at early pregnancy was largely complete from 1992 onward.

^d^ This analysis was restricted to individuals born during 1987-2008 because information on maternal diabetes was available from 1987 onward.

^e^ Low Apgar score was defined as Apgar score <7.
